# Spotting the difference between pairs of nearly identical Perlin images: Influences of presentation formats

**DOI:** 10.1371/journal.pone.0264621

**Published:** 2022-02-25

**Authors:** Leo Poom, David Fällmar

**Affiliations:** 1 Department of Psychology, Uppsala University, Uppsala, Sweden; 2 Department of Surgical Sciences, Radiology, Uppsala University, Uppsala, Sweden; Tsinghua University, CHINA

## Abstract

We investigated human performance in speed and precision of detecting a deviating visual target embedded in one of two otherwise identical non-figurative Perlin-noise images (i.e. a spot-the-difference task). The image-pairs were presented in four different presentation formats: spatially separated in horizontal or vertical direction while simultaneously presented, or sequentially separated on the same location with a brief delay or without any delay. In the two spatial conditions failure to detect the target within 30 sec (change blindness) occurred in about 6–7% of the trials, and with the brief delay 2.4% of the trials. Fast error-free detection (i.e. pop out) was obtained using the sequential format with no delay. Average detection time when target was detected was about 9 sec for the two spatial formats. Detection time was faster, about 6 sec, for the brief delay condition. In trials where detection was reported, the precision of locating the target was equal in the horizontal and brief delay conditions, and better than in the vertical condition. Misses obtained in the horizontal and brief delay conditions were also more strongly correlated than correlations between misses in the vertical and horizontal, and between the vertical and brief delay conditions. Some individuals’ performances when comparing images in the vertical direction were at chance level. This suggests influences of known poorer precision when making saccades in the vertical compared to horizontal direction. The results may have applications for radiologists since the stimuli and task is similar to radiologists’ task when detecting deviations between radiological images.

## Introduction

We investigated performance in detecting a difference between two images depending upon presentation format. A change detection technique was used with stimuli composed of pairs of nearly identical images containing a single local difference target (i.e. a spot-the-difference task). The images consisted of pseudo-random non-figurative luminance noise where nearby luminance values of pixels were highly correlated, so-called Perlin noise [1]. A randomly located deviation of luminance (the target) added to one of the images created a local difference between image pairs. This research was inspired from noting that radiologists use mixed formats in the arrangement and display of radiological images, either separated in the horizontal or vertical direction, when looking for deviations. The purpose of the target and pair-wise comparison was therefore to use images that mimic radiological images with added lesions, to address both theoretical and practical concerns.

The image-pairs were presented in four different presentation formats: presented simultaneously separated in horizontal [1] or vertical [2] direction, and repeatedly on the same location with a brief delay [3] or without a brief delay [4] between images. Detecting the target in simultaneous presentation formats involve making saccades back and forth between images (side to side or up and down), in contrast to the sequential conditions with a brief delay. These three conditions require using iconic or short-term visual memory in the comparison process. Such task involves focused attention and is subject to change blindness where large changes may be unnoticed [2]. The fourth condition, sequential with no delay, results in a “pop-out” experience of the target and involves pre-attentive mechanisms.

Performance in spotting differences across visual space may differ depending on whether simultaneously presented images are horizontally or vertically separated, or if the pairs are presented sequentially on the same location with a small inter-stimulus interval (ISI). Neurobiological differences between vertically and horizontally directed saccades are a potential cause of such differences. The asymmetries between vertical and horizontal saccades consists in that distractors interfere less with horizontal saccades [3], horizontal saccades results in less undershoot [4], and horizontal saccades before recall specifically enhances subsequent memory performance [5]. Another possible reason to expect differences in performance across horizontal and vertical presentation formats is that we are more familiar with visual search along the horizontal direction, i.e. along the ground, on tables etc., than in the vertical direction.

Besides being theoretically interesting, this research has important applications for radiologists who routinely compare radiological images to detect differences between images taken at different time points. In fact, there is no consensus recommendation available that describe what presentation format to use when comparing such images. In clinical radiology, using display protocols with vertical comparisons have practical advantages, such as when two MRI scans with several different sequences are to be compared. A personal experience is that some radiologists insist on using display protocols with horizontal comparisons. This observation inspired two hypotheses; that horizontal comparisons are superior and/or that some individuals are less proficient in performing vertical comparisons.

Perlin-noise images were chosen as visual stimuli for this study since they contain similar image elements as radiological images, but are available for well-defined and controlled adjustments.

To our knowledge this is the first time influences of presentation format on change detection performance and change blindness are investigated. The study also introduces the Perlin noise generating technique in perception research.

## Method

### Stimuli

**Fig 1:**
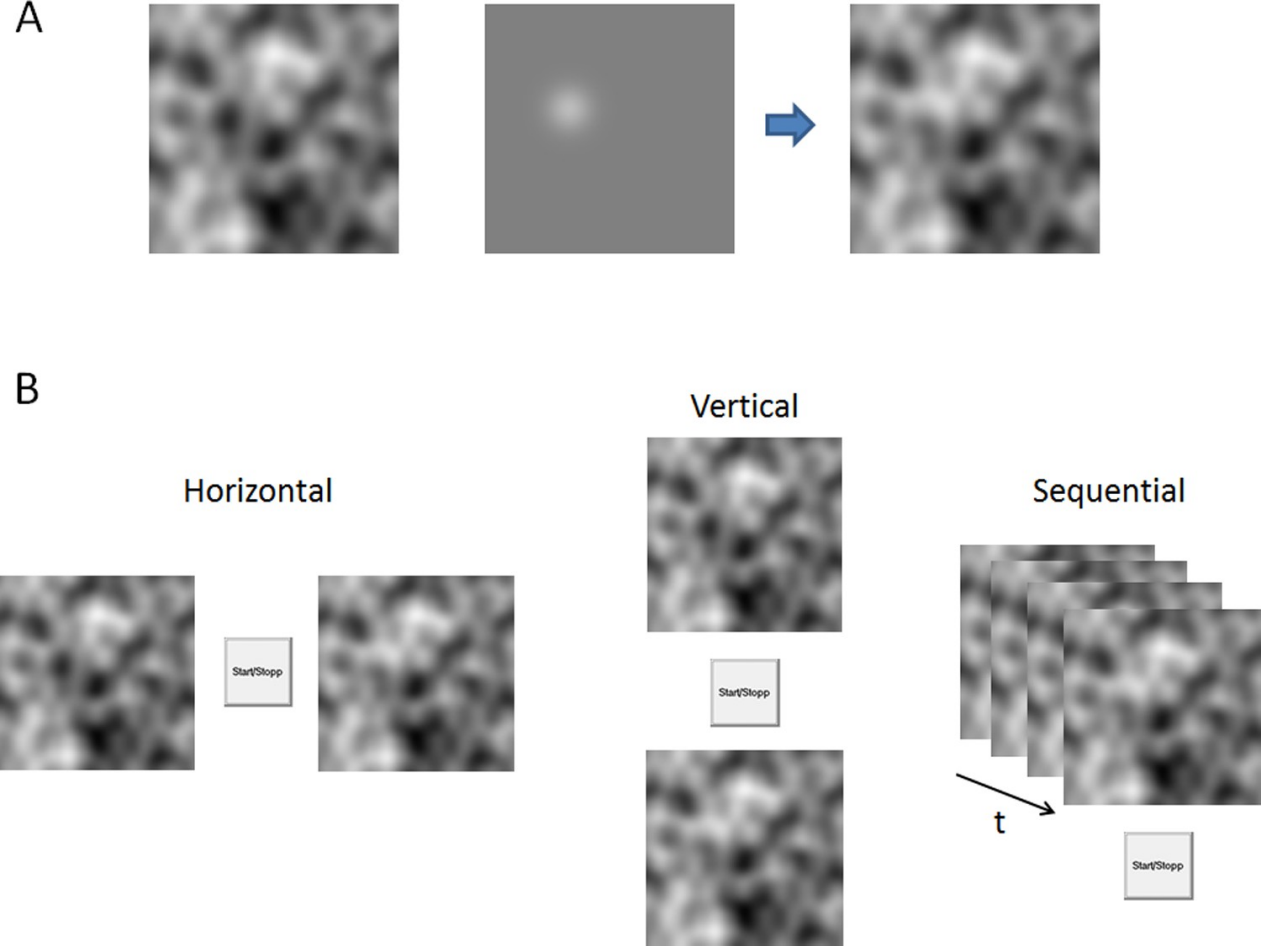
Stimulus and presentation formats. (A) An example of a Perlin pattern on the left, and a randomly positioned Gaussian blob superimposed on a homogenous background in the middle. To create the stimuli pairs the blob was added to the Perlin pattern as shown in the rightmost image. (B) Illustration of the different conditions with the response button and the image pairs separated horizontally, vertically, and overlaid with repeated sequential presentation over time t. In the two sequential conditions each image was then presented 400 milliseconds with ISI (inter stimulus intervals) = 0 or ISI = 200 milliseconds.

Perlin [1] developed an award winning technique used to produce natural appearing pseudo-random textures on computer generated surfaces for visual effects in movies, which has been further developed since then. Here, the original Perlin noise generating algorithm from 1985 is used to create the stimulus patterns. In such patterns nearby luminance values are correlated as in typical visual images, as opposed to random dot images where the luminance values of all pixels are completely uncorrelated. These correlated patterns resemble discrete relevant lesions in radiological images, such as intraparenchymal metastases in a computed tomography image. Briefly, these patterns are created by defining a grid of random gradient vectors at the corners of each square grid covering the stimulus area. Then the dot product between the gradient vectors and their offsets to each pixel within the grid square are computed, and finally these values are smoothly interpolated to obtain the luminance value at that pixel. For the present purpose 30 square shaped images were created, each with a side 8.7 cm. Each image contained a 3 x 3 grid to determine the locations of the random vectors. The original Perlin algorithm produce some images with directional artefacts, these were visually sorted out. An example of a resulting Perlin noise image is demonstrated in the leftmost image in Fig 1A.

The 30 images were doubled to create identical pairs, and to one of the images in a pair a randomly located target deviation was added. The target was created by adding a 2D Gaussian luminance distribution to the Perlin image. The Gaussian had a width σ = 3.5 mm resulting in a visible deviation between otherwise identical image pairs (the target) with a diameter about 2 cm. If the luminance of the Perlin pattern at the location of the target was lower than the average luminance (R = G = B = 128) then a target with positive contrast was added (otherwise a negative contrast with negative luminance values of the target was added) to create each stimulus pair. All images were also visually inspected to make sure that the target was undetectable from any image alone. The middle image in Fig 1A shows an example of a target with positive contrast added to a homogenous luminance field (the grey field has R, G, B values set to 128, which is the same as the average luminance across the Perlin noise images). The rightmost image is the result of adding the target to the Perlin image.

The four presentation formats, or conditions, were horizontally separated images, vertically separated images, and sequential repeated presentations of the two images with or without a delay (Fig 1B). The separation between image-pairs in the horizontal and vertical conditions was 3 cm. In the sequential conditions each image was shown for 400 milliseconds and then after an inter stimulus interval (ISI) of 0 or 200 milliseconds replaced at the same position with the other image in the pair. In other words, the sequential images were exchanged with or without a pause (showing no image). This was repeated until participants indicated detection of the target. The following demos, movies ISI=200 (S1 Movie) and ISI=0 (S2 Movie), demonstrate sequential presentation of identical image pairs with one deviating target presented sequentially with ISI = 200 milliseconds and ISI = 0 respectively.

Image pairs with three signals strengths of the target were created (peaks of the luminance distribution with red, green, and blue, or RGB values all set equal to 40, 50, or 60). The peaks of the luminance distributions of the target added to the Perlin images were below the level where the targets would not pop out from single images. The stimulus set consisted of 30 pairs of images, 10 of each of the three signal strengths. These same 30 image pairs were used in all four presentation formats. A computer program was developed to create the Perlin images and add the target on a random position within the image, and another computer program was developed for stimulus presentation, response collection and storage of the data. The software used was Visual Basic 5 (VB5). The stimuli were presented on a 17 inch screen with 60 Hz refresh rate.

### Procedure

Participants were seated with a viewing distance of 60 cm from the screen, so 1 cm on the screen corresponds to approximately 1° of visual angle. At each trial participants were instructed to first detect the target and click the response button located between the images or below the sequentially presented images. The detection time was measured and stored as a performance result. Immediately after the first response one of the images disappeared (this was always the left image in the horizontal condition and the bottom image in the vertical conditions) and the other image remained visible on the screen. The stimulus generation procedure by which targets could be detected only by comparing image pairs made it impossible to detect the target from single images of the image pairs. So, irrespectively which image stayed on the screen, the target had to be located from when both images were visible or from the last switch between the images before detection was reported. A maximum of 30 seconds was allowed for the participants to search for the target deviation.

If the target was detected within the time limit, a second task was to indicate the location of the target by moving the cursor to that location in the remaining image and click the mouse whereby a dot appeared and the distance between the marked location and the target was stored as a second performance result. At the third click on the response button the next trial was initiated and the procedure was repeated.

The horizontal, the vertical and ISI = 200 conditions were presented in four blocks of 30 trials each. The vertical, horizontal, and ISI = 200 blocks were presented in balanced order between participants, whereas the ISI = 0 condition was presented last. The ISI = 0 condition was included as a comparison involving pre-attentive search since the target effortlessly attracted attention and popped out from the background. Participants could take a break after any trial.

### Participants

All 30 participants (14 women) had normal or corrected to normal visual acuity, ages between 20 and 71 years. They were informed that their identity would not be revealed and that they could cancel the experiment at any time. The sample size was partly based on experience from previous experiments in this field where similar or smaller samples have been used, and that the expected effect sizes were relatively large [20]. Our expectations of obtaining large effect sizes were fulfilled. Verbal consent to participate in this study was obtained from each participant. Participants could leave the experiment with no consequences. The study was approved by the Institutional Review Board in Uppsala and was conducted in accordance with the Declaration of Helsinki.

### Statistics

Both traditional frequentist p-values and Bayes factors are presented. Statistical tests were performed by the freely available statistical software JASP [6] (freely available at https://jasp-stats.org/download/). The Bayes factor BF_10_ is the ratio between the probabilities of the results given H_1_ and H_0_ and is thus a measure of the relative evidence in favour of H_1_ and ranges from 0 to infinity (the relative evidence in favour for H_0_ is then BF_01_ = 1/BF_10_). Bayes factors are more intuitive than p-values and have several other advantages [7]. For example, unlike the p-value, the Bayes factor can be interpreted as strong evidence in favour, or against, the null hypothesis (H_0_). All reported Bayes factors were computed using the default settings in JASP for the effect size priors. As a guideline it has been suggested that BF_10_ (BF_01_) between 1 and 3 (1-1/3) are barely worth mentioning, from 3 to 10 (1/3–1/10) are considered moderate evidence, from 10 to 30 (1/10–1/30) as strong evidence, between 30 to 100 (1/30–1/100) as very strong, and beyond that extremely strong evidence [8].

Additional analyses, as described in the text, were performed by the following on-line calculators. Comparison of proportions calculator used the "N-1" Chi^2^ test https://www.medcalc.org/calc/comparison_of_proportions.php as recommended by Campbell [9]. The difference of two correlation coefficients obtained from the same sample, with the two correlations sharing one variable in common was analysed using the online calculator at http://quantpsy.org/corrtest/corrtest2.htm as suggested by Lee and Preacher [10].

We decided to use a radius r = 1 cm around the target centre to discriminate between hits and misses in locating the target with the cursor. This area of interest (AOI) captured the visible deviation of the target which had a diameter of about 2 cm and was large enough to allow some varying precision when placing the cursor on detected targets. Chance level performance in placing the cursor within the AOI was calculated by comparing the AOI area to the whole image area. The Perlin images were 8.7 cm x 8.7 cm = 75.69 cm^2^. The surface area of the AOI is then 2 · pi · r^2^ = 2 · pi ≈ 6.28 cm^2^. A randomly chosen location for the cursor would result in an expected probability 6.28/75.69 ≈ 0.083 of a hit within the AOI (8.3% hits or 91.7% misses). A statistically significant percentage of hits is obtained if the number of misses is below 24 out of 30 trials or below 80%, using α-value = .05 as calculated using the Binomial distribution. A Bayes factor BF_10_ > 10 (considered to be strong evidence) is obtained if the number of misses is below 22 out of 30 trials, or below 73%.

## Results

### Individual misses

**Fig 2:**
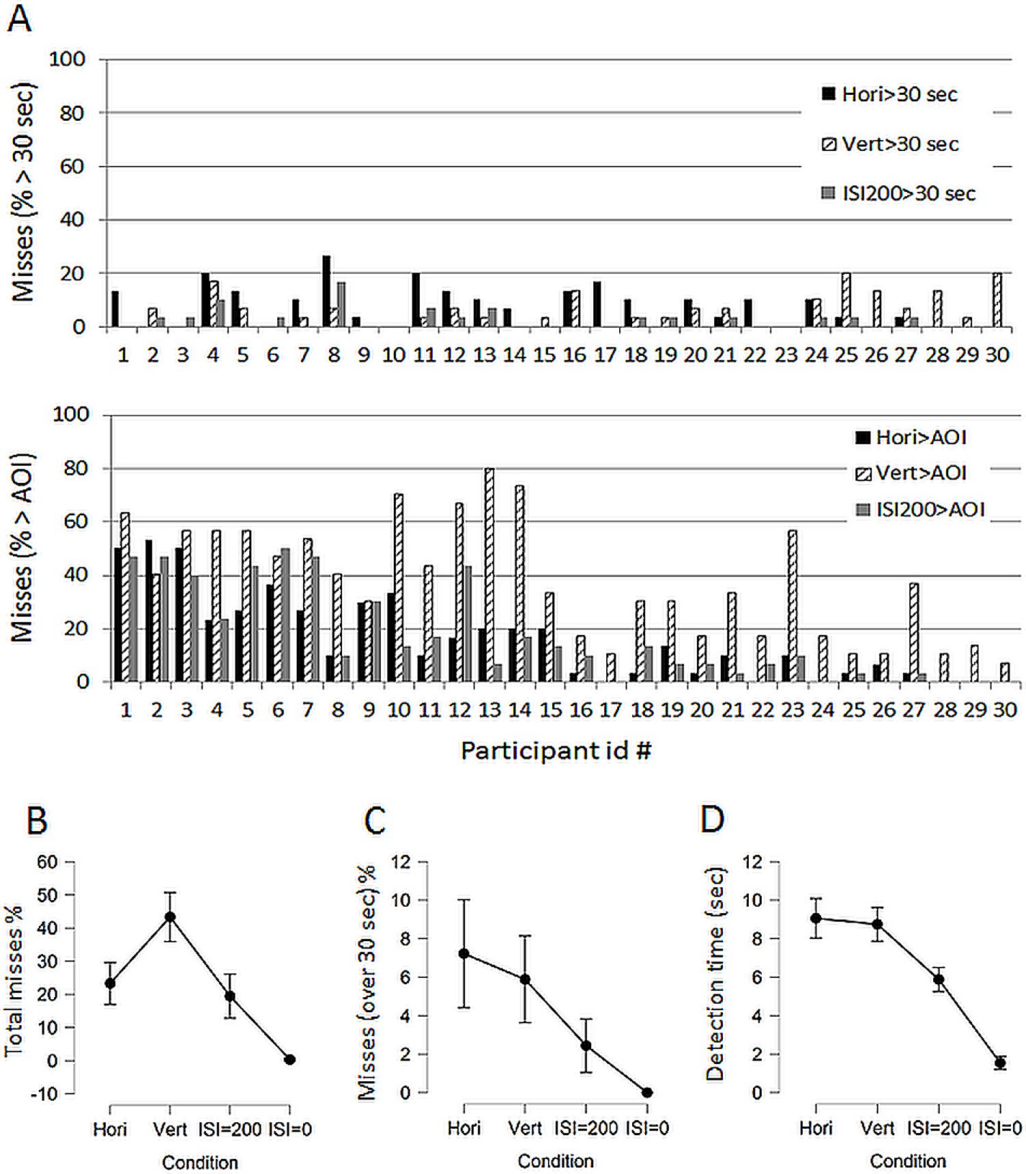
Individual and group performance. A. Individual percentage of misses made in the horizontal, vertical, and ISI = 200 conditions. Top: misses due to failure to detect any deviation within the 30 sec time limit (Change blindness). Bottom: misses due to marking outside the AOI (that is > 1 cm from the target) when detection is reported. Individuals in the graphs are sorted contingent on the total number of misses (sum of failed detections within 30 sec and markings outside the AOI) in the horizontal condition. (B) The group level average total percentage of misses is displayed where no target was detected within the 30 seconds time limit or the markings were made outside the area of interest (AOI). (C) The group level misses consisting of no target detected within 30 seconds, i.e. change blindness, are selectively shown. (D) Detection times in seconds for trials were the markings were made within the AOI (< 10 mm from the target centre). The 95% CI are displayed.

The percentage of trials where the participants failed to detect any difference between image pairs within the 30 seconds limit are shown in the top graph in Fig 2A (not showing the ISI = 0 condition since no participant failed to find the deviation within 30 seconds). The percentage of trials where participants failed to place the cursor within the AOI is shown in the bottom graph in Fig 2A (not showing the ISI = 0 condition since there were only three total failures in this condition). In the vertical condition there were a few participants who failed to reach reliable performance above chance level.

### Group data: Misses

Fig 2B shows the percentage of total misses where participants failed to detect any target within the 30 seconds limit, or marked the perceived location of the target outside the AOI. Trials where participants indicated detection but failed to direct the cursor within the AOI could be correct detections followed by failure to hit the AOI, or result from change blindness. On average, participants missed 43% of all trials in the vertical presentation condition, 23% in the horizontal, 19% in the ISI = 200 condition, and only 0.3% in the ISI = 0 condition. The main effect of condition was statistically significant (F(3, 87) = 70.3, p < .001; BF_10_ = 4.2·10^20^). Post hoc p-values and Bayes factors for pairwise differences were calculated and the only difference that was non-significant (or anecdotal in Bayesian jargon) was between horizontal and ISI = 200 conditions (t(29) = 1.3, p = .19; BF_10_ = 2.5). All other differences were significant (p-values < .001; BF_10_’s > 10 000).

Fig 2C shows the subset of all trials where participants failed to detect any target within the 30 seconds time limit. These trials could therefore be classified as true change blindness. For the horizontal and vertical conditions on average 7% and 6% of all trials could be classified as change blindness. In the sequential ISI = 200 and ISI = 0 conditions change blindness occurred in only 2.4% and 0% of all trials, respectively. The main effect of condition on change blindness occurrences was significant (F(3, 87) = 14.0, p < .001; BF_10_ = 240 000). Post hoc tests showed that the only difference in change blindness occurrences that was non-significant (or providing weak support for the null model in Bayesian jargon) was that between the horizontal and vertical condition (t(29) = 1.07, p = .29; BF_10_ = .25). All other differences were significant (p’s between .02 and < .001; BF_10_’s between 5.7 and 2100).

### Group data: Time until successful detection

Detection times for successful detections (including only trials where the cursor was set within the AOI) is shown in Fig 2D. The main effect of condition is statistically significant (F(3, 87) = 151.0, p < .001; BF_10_ = 2.6·10^33^). Post hoc tests show no difference between detection times in the horizontal and vertical condition, and the BF provides moderate evidence for the null model (t(29) = .77, p = .44; BF_10_ = .24), all other differences in detection times were significant (p values < .001; BF_10_’s between 39000 and 3·10^14^).

### Relations between presentation formats

The differences between total misses from each pair of conditions (horizontal, vertical and ISI = 200) for each participant were analysed using the online available Comparison of proportions calculator by the "N-1" Chi^2^ test https://www.medcalc.org/calc/comparison_of_proportions.php as recommended by Campbell [9]. This analysis at the individual level can be considered as 30 repeated experiments which increase the reliability and provide finer grained information than single analyses performed on group averages. For 12 out of 30 participants (40%) the difference between total misses obtained in the vertical and horizontal presentation formats were statistically significant (all with more misses obtained in the vertical condition), with p-values varying from p = .02 to p < .0001. All these 12 also obtained significant differences in misses between the vertical and ISI = 200 condition. In a total of 16 out of 30 participants (53%) the difference between the vertical and ISI = 200 condition were statistically significant (all with more misses obtained in the vertical condition), with p-values varying from p = .03 to p < .0001. Only one participant had statistically significant difference in total misses between the horizontal and ISI = 200 condition (≈ 3%), with zero errors in the ISI = 200 condition and 17% errors in the horizontal condition, p = .019. For a table displaying the differences for each individual between total misses from each pair of conditions, see S1 Table.

**Fig 3:**
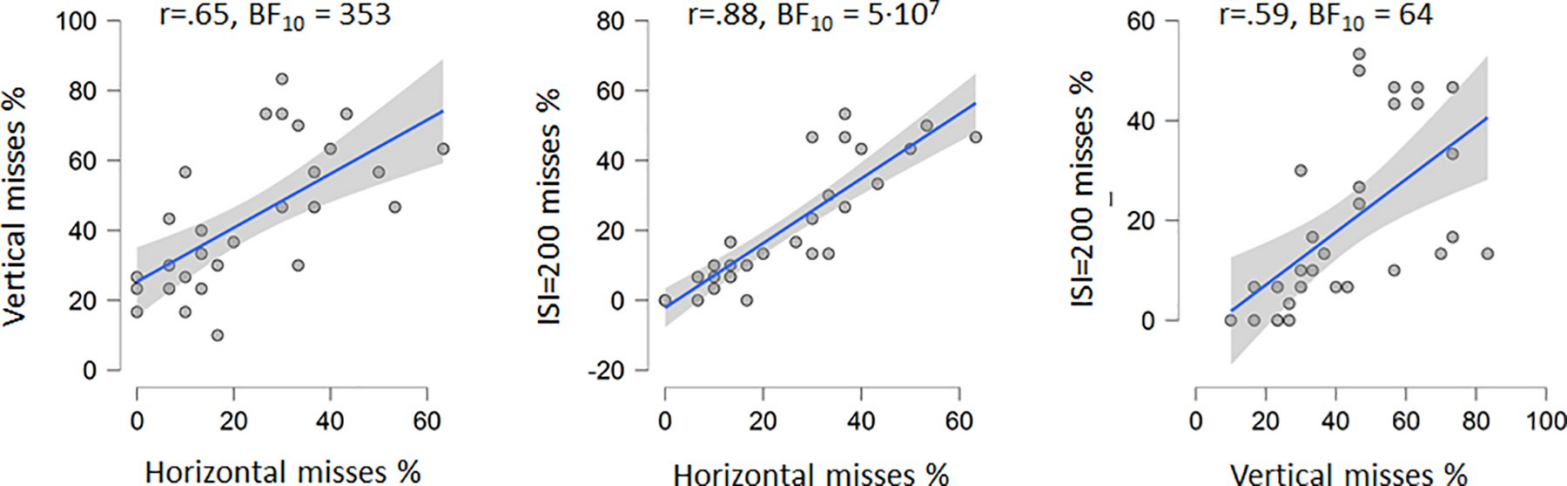
Scatterplots. The Pearson correlations analyses between total misses obtained in the horizontal, vertical, and the sequential condition with ISI = 200 milliseconds. Correlations, Bayes factors, regression lines and the 95% CI are shown. All p values < .001.

In addition, from the bottom graph in Fig 2A it seems that the misses obtained from the horizontal and ISI = 200 millisecond conditions co-vary more than horizontal and vertical, or the vertical and ISI = 200 conditions. Fig 3 shows the scatterplots with correlations and Bayes factors to investigate this co-variation more closely. Correlations between the total misses obtained in the three conditions were all statistically significant (all p values < .001). The difference of two correlation coefficients obtained from the same sample, with the two correlations sharing one variable in common was analysed using the online calculator at http://quantpsy.org/corrtest/corrtest2.htm [10]. Differences were obtained for Hori-vert vs. hori-ISI200 correlations (2-tailed p = .010), and for Hori-ISI200 vs. Vert-ISI200 correlations (p = .00084), but not for the Hori-vert vs vert-ISI200 correlations (p = .38). These results suggests that search in the horizontal and ISI = 200 conditions involve more similar strategies/mechanisms than search in the vertical condition.

Raw data is provided in S1 Data.

## Discussion

The study shows that the display format influences performance in visual perception comparisons. The novel change detection task using Perlin noise images involves both pre-attentive and focused attention search mechanisms depending on the signal strength of the luminance target. Local change (luminance onset/offset, or motion) in a scene typically direct attention reflexively towards the change resulting in a pop-out effect, but in the absence of local change signals or when a blank period or a saccade happen during the change, attention is guided to areas of high-level interest whereby a serial search process takes place. Of course, if the target is very bright compared to the background it will pop-out even when a blank period or a saccade intervene the change. Here the strength of the target was chosen to activate focused attention in these conditions to investigate the influence of presentation formats on change blindness. The target was effortlessly detected and visually popped out when no delay was presented between images but become effortful with a brief delay of 200 milliseconds, which is the hall-mark of inattentional blindness due to the involvement of the bottleneck of focused attention. This can be relevant to optimize perceptual performance in an applied context, such as radiology. The difference between horizontal and vertical display mimics the optional display formats of comparisons between current and prior scans in all radiological practice. The particular difference between the sequential comparisons with or without delay can to some extent resemble the difference between thin slices versus 5 mm reconstructions, when scrolling through an image volume of a computed tomography scan.

The results show that individuals occasionally failed to find the target within the 30 second time limit. These trials were classified as “true” change blindness trials. Less misses occurred in the sequential condition with ISI = 200 millisecond (on average 2.4%) than in the horizontal and vertical conditions (7% and 6%). Thus, the numbers of change blindness trials were similar in the horizontal and vertical conditions. As expected, in the ISI = 0 condition involving pre-attentive processes, no failures to detect the target within 30 seconds were found. Trials where participants reported that a target was detected, but failed to place the cursor within the AOI could be a result of true change blindness trials where participants guessed or misperceived the location of the target, or correct detections but failures in directing the cursor. Targeting outside the AOI was, however, very rare in the ISI = 0 condition (0.3%), providing evidence that the AOI misses in the other conditions were not caused by difficulties to direct the cursor to the target. It simultaneously provides evidence that the likely explanation of the AOI misses were due to misperceptions or guesses rather than low precision in placing the cursor on the centre of a correctly perceived target. When detection was reported in the vertical condition participants indicated the location of the target outside the AOI with about twice the frequency (43%) compared to the horizontal (23%) and ISI = 200 conditions (19%). This can have direct applications in all visual comparison tasks where optimal performance is sought. One hypothesis of this study was that human perception is more adapted to horizontal comparisons, rendering it a superior perceptual strategy compared to vertical. This hypothesis is at least partly supported by the results. The current findings present an opportunity to conduct future studies with trained radiologists as subjects, using real radiological images.

If the same search strategy was employed in separate conditions we would expect higher correlations between these performances than between conditions where different search strategies were used. The present data indicates that correlations in performances, in terms of total misses, in the horizontal and ISI = 200 conditions were stronger than between the correlations between the horizontal and vertical conditions, and between the vertical and ISI = 200 conditions. This is evidenced by comparing the Bayes factors and the correlations or shared variances (77% shared variance between the horizontal and ISI = 200 conditions compared to 43% and 35% shared variance between horizontal and vertical and between the vertical and ISI = 200 conditions respectively). In addition, individual data showed that the individual misses in horizontal and ISI = 200 conditions were similar for 29 participants, only for one participant was the number of misses significantly different between these two conditions (as would be expected with repeated experiments and an α-level = .05). The number of misses in the horizontal and vertical conditions differed significantly for 12 out of 30 individuals, and between the vertical and ISI = 200 condition the number of misses differed significantly for 16 out of 30 individuals. The results provide evidence for different search strategies between the vertical and horizontal conditions as well as between the vertical and ISI = 200 condition. No evidence to support any such difference between the horizontal and the ISI = 200 conditions were found.

Thus, about half of the participants showed poor performance in the vertical condition compared to the horizontal and ISI = 200 condition, a few participants even failed to reach reliable performance above chance level. This may indicate that these individuals have especially inefficient visual strategies when comparing images in the vertical direction, possibly due to inefficient vertical saccades. This finding supports the second hypothesis; that some individuals are more proficient and perform better using horizontal rather than vertical comparisons.

Similar performance and higher correlations between total misses in the horizontal and the sequential ISI = 200 conditions compared to the vertical and horizontal and the vertical and ISI = 200 conditions suggests similar search strategies or processes. The vertical condition may also involve additional distracting components, such as lower precision in vertical saccades. To search for the target in horizontal and vertical displacements of images require repeated horizontal and vertical saccades which are mediated by distinct processes. Vertical saccades show more interference from a distractor than horizontal saccades [3], and results in more hypometric deviations, or undershoot, when directed to targets compared to horizontal saccades [4]. In addition, actively engaging in horizontal eye movements before recall enhances subsequent memory performance. This last peculiar phenomenon is called saccade-induced retrieval enhancement (SIRE) and there is evidence that it results from interhemispheric interactions stimulated by horizontal saccades, but not vertical saccades [5]. Another possible explanation, which could be related to the vertical versus horizontal saccade asymmetry, may be that we typically have more experience of making visual search and comparisons along the horizontal than along the vertical. This is simply due to gravity, we live on a flat ground where objects lay around on the horizontal ground level, on floors or tables and are rarely separated along the vertical dimension. This gravity constraint also leads to more experience in making horizontal saccades than vertical saccades, therefore the saccade asymmetry and gravity-based explanations may be related.

Our results may also have implications on computerized image analyses, which have become very popular lately due to the great progress achieved in applied artificial intelligence (AI in the form of deep learning). This research has also shed new light on the strengths, weaknesses, constraints, and differences between human cognition and AI. In dermatology and radiology, AI applications are already in clinical use [11, 12]. Although there are headlines claiming how the latest AI has outperformed humans, surprisingly few studies compare the performance of humans and AI, from the sparse available data. However, it seems that the accuracy of deep learning algorithms is equivalent to health-care professionals (for a review see [13]), but falls short in other aspects. For example, AI applications are narrowly focused on achieving a specific task [12], lack the human assessors ability to generalize [14], and lack the human ability to deal with noise and image artefacts [15]. Another challenge for machine learning and especially for radiological applications and research is a general shortage of publicly available labelled data [16]. Although the task of detecting a target in otherwise identical Perlin image pairs would be a trivial task for an algorithm (just take the difference between luminance levels at each image location), an additional Perlin noise mask (with specified luminance variation) can be added to one of the images to make the task non-trivial even for machine vision. This could provide a means to generate a large number of artificially created images as required for research on machine learning in clinical settings.

The radiologists’ task to detect deviations in images is similar to the task employed here and the Perlin images are similar to CT or MR images and X-ray scans. In radiology, a majority of diagnostic errors are perceptual [17–19]. Radiologists are also subject to misperceptions and inattentional blindness. Drew et al. [20] let skilled radiologists perform a lung nodule detection task and when they inserted a gorilla much larger than the average nodule in the image, 83% of radiologists failed to detect it even though they looked directly at the location of the gorilla. Brams et al. [21] demonstrated differences in eye movements between different levels of experience in interpreting chest X-rays, and accompanying differences in accuracy between more experienced and less experienced participants, and suggested that experts use more holistic search strategies involving support from long-term memory. Here, participants were non-experts, and whereas radiologists typically view each image on a separate monitor so the images are larger and separated by larger distance here the images were presented on the same monitor and smaller. Hence, our results may not be generalizable to experienced radiologists. Still, understanding visual search performances using different presentation formats can help to reduce the number of errors in pathology detection. In clinical practice, there is no consensus regarding how radiological images should be displayed to optimize diagnostic performance. Vertical pair-wise comparisons are not uncommon, and future studies are needed to establish the specific effect of presentation format on diagnostic performance in an applied context. The method presented here to create quasi-random stimuli provides an efficient tool for these purposes using non-figurative quasi random patterned images, as opposed to procedures generating such images from figurative photographs [22].

## Open practices statement

All data are available by request from the author leo.poom@psyk.uu.se.

## Supporting information

S1 Movie ISI = 200 msec. Movie sequence showing the sequential presentation format with 200 msec inter stimulus interval.
(MP4)

S2 Movie ISI = 0 msec. Movie sequence showing the sequential presentation format zero inter stimulus interval (otherwise same sequence as S1).
(MP4)

S1 Table Differences between presentation formats. Inference tests of the individual differences of total misses between conditions, displaying p-values for the statistically significant differences (α = .05), ns = non-significant.
(DOCX)

S1 Data (XLSX)
