# Supplementary material for: Spotting the difference between pairs of nearly identical Perlin images: Influences of presentation formats
Source: PLoS One. 2022 Feb 25;17(2):e0264621. doi: 10.1371/journal.pone.0264621 (PMC8880654; doi:10.1371/journal.pone.0264621)
Supplement: S1 Table — Inference tests of the individual differences of total misses between conditions, displaying p-values for the statistically significant differences (α = .05), ns = non-significant. (DOCX) [file pone.0264621.s003.docx]

| Participant | Hori vs. Vert | Hori vs. ISI=200 | Vert vs. ISI=200 |
| --- | --- | --- | --- |
| 1 | ns | ns | ns |
| 2 | ns | ns | ns |
| 3 | ns | ns | ns |
| 4 | .02 | ns | .002 |
| 5 | ns | ns | ns |
| 6 | ns | ns | ns |
| 7 | ns | ns | ns |
| 8 | ns | ns | ns |
| 9 | ns | ns | ns |
| 10 | .0045 | ns | <.0001 |
| 11 | ns | ns | ns |
| 12 | .001 | ns | .042 |
| 13 | <.0001 | ns | <.0001 |
| 14 | .0003 | ns | <.0001 |
| 15 | ns | ns | .033 |
| 16 | ns | ns | ns |
| 17 | ns | .019 | ns |
| 18 | ns | ns | ns |
| 19 | ns | ns | .032 |
| 20 | ns | ns | ns |
| 21 | .019 | ns | .0028 |
| 22 | ns | ns | ns |
| 23 | .0001 | ns | .0001 |
| 24 | ns | ns | .012 |
| 25 | .023 | ns | .023 |
| 26 | ns | ns | .0056 |
| 27 | .0014 | ns | .0014 |
| 28 | .0056 | ns | .0056 |
| 29 | .019 | ns | .019 |
| 30 | .003 | ns | .003 |
